# Seoul Virus Infection Acquired at Private Pet Rat Breeding Facility, Germany, 2024

**DOI:** 10.3201/eid3110.250362

**Published:** 2025-10

**Authors:** Fabian Baalmann, Mario Hönemann, Stephan Drewes, Martin Eiden, Calvin Mehl, Dennis Tappe, Birte Pantenburg, Steffi Bellmann, Ingrid Möller, Melanie Maier, Corinna Pietsch, Rainer G. Ulrich, Johannes Münch

**Affiliations:** University of Leipzig Medical Center, Leipzig, Germany (F. Baalmann, M. Hönemann, M. Maier, C. Pietsch, J. Münch); Friedrich-Loeffler-Institut, Greifswald-Insel Riems, Germany (S. Drewes, M. Eiden, C. Mehl, R.G. Ulrich); Deutsches Zentrum für Infektionsforschung, Partner site Hamburg-Lübeck-Borstel-Riems, Greifswald-Insel Riems (C. Mehl, R.G. Ulrich); Bernhard Nocht Institute for Tropical Medicine, Hamburg, Germany (D. Tappe); University of Leipzig Institute of Social Medicine, Occupational Medicine, and Public Health, Leipzig (B. Pantenburg); City of Leipzig Public Health Office, Leipzig (B. Pantenburg, S. Bellmann, I. Möller); University Medicine Mannheim Medical Clinic for Nephrology, Kidney Transplantation, Endocrinology, Rheumatology, and Pneumology, Mannheim, Germany (J. Münch)

**Keywords:** Seoul virus infection, hantaviruses, viruses, zoonoses, Germany, acute kidney injury, orthohantavirus, rats

## Abstract

We report a case of severe acute kidney injury in a patient in Germany infected with Seoul virus. Clinical and laboratory analysis linked the infection to a pet rat breeding facility in central Germany. Increased surveillance and a One Health approach are needed, given the rising popularity of pet rats.

Hantaviruses, a diverse group of zoonotic pathogens carried primarily by rodents, can cause hemorrhagic fever with renal syndrome (HFRS) and hantavirus cardiopulmonary syndrome (*1*). In Europe, Puumala virus and Dobrava-Belgrade virus are the most prevalent human pathogenic hantaviruses (*2*). Seoul virus (SEOV) (species *Orthohantavirus seoulense*) is the only hantavirus with a worldwide distribution. Norway rats (*Rattus norvegicus*), black rats (*R. rattus*), and related species represent the main natural reservoir; however, pet rats recently have emerged as an important source of SEOV infections (*3*). The clinical spectrum of SEOV infection in humans ranges from mild febrile illness to HFRS-like disease that can include fever, hepatitis, and gastroenteritis, and, less commonly, kidney involvement (*4*,*5*). In contrast to its worldwide distribution, autochthonous SEOV infections in Germany remain rare; only a few cases have been reported to date (*4*,*6*).

## The Study

In March 2024, a previously healthy 44-year-old woman without any underlying conditions sought care at the nephrology clinic at University Hospital Leipzig (Leipzig, Germany) with a 6-day history of fever, fatigue, diarrhea, and hypotension. Five weeks before symptom onset, the patient visited a private pet rat breeding facility and subsequently acquired rats for her children. Initial laboratory workup revealed thrombocytopenia, mildly elevated transaminases, and acute kidney injury (Appendix Figure 1, panel A). Urinalysis revealed hematuria and nephrotic-range proteinuria (urine protein–creatinine ratio >6 g/g [reference range <0.07 g/g]). Renal ultrasound showed enlarged, edematous kidneys (Appendix Figure 1, panel B), mild ascites, and splenomegaly. We observed no sonographic sign of chronic kidney disease or liver pathology. Given the deteriorating kidney function (peak serum creatinine was 14.3 mg/dL [reference range 0.67–1.18 mg/dL]), we initiated acute hemodialysis and performed a kidney biopsy.

**Figure 1:**
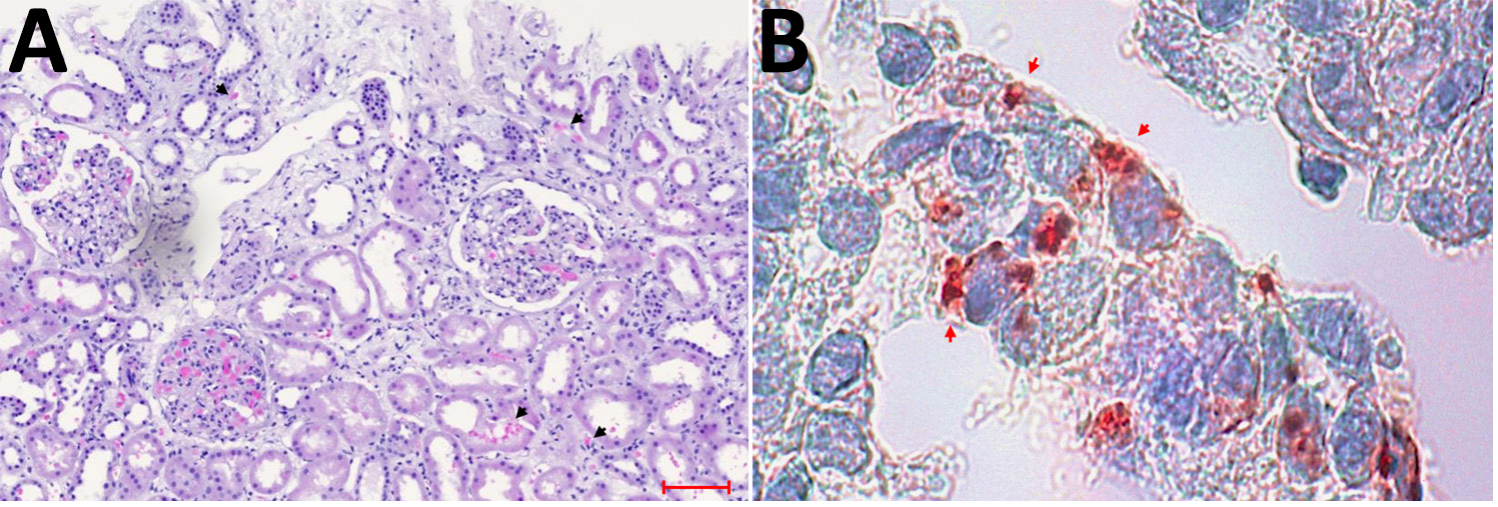
Light microscopic analysis and immunohistochemical staining results of index patient’s kidney biopsy indicating severe Seoul virus infection with acute kidney injury, Germany, March 2024. A) Light microscopy of the hematoxylin- and eosin-stained biopsy demonstrating severe acute tubular injury with interstitial and tubular bleeding (black arrow heads), tissue edema, and interstitial inflammation. Scale bar indicates 100 μm. B) Immunohistochemical staining (red) using mouse monoclonal antihantavirus antibody (Progen Biotechnik, https://www.progen.com) and the DCS-AEC chromogen kit (Agilent–DAKO, https://www.agilent.com) showing hantavirus antigen in renal tubular cells (red arrow heads). Original magnification ×100.

Histopathologic examination revealed severe acute tubular injury with interstitial hemorrhage, edema, and inflammation (Figure 1, panel A). Only 2 of 21 glomeruli were sclerotic, and we observed minimal interstitial fibrosis with tubular atrophy. Specific immunohistochemical staining for hantavirus antigen was positive within the tubular cells and the tubulointerstitial space (Figure 1, panel B). Concurrently, lineblot assays performed by using recomLine HantaPlus (Mikrogen Diagnostics, https://www.mikrogen.de) of admission serum samples demonstrated positivity for hantavirus IgG and IgM, supporting the diagnosis of hantavirus-associated nephritis. Reactive bands included Hantaan virus antigen for IgG and Puumala, Hantaan, Dobrava-Belgrade, and Seoul virus antigens for IgM (Appendix Table 1). To further delineate the infecting hantavirus, we used a panhantavirus reverse transcription PCR targeting a 412-nt region of the viral large segment (*7*) on blood and urine samples. The subsequent sequence analysis and comparison to GenBank entries confirmed an infection with SEOV.

After 3 hemodialysis sessions (hospital days 5–7), the patient’s clinical condition improved; we observed normalization of platelet counts by day 3, transaminase activities by day 6, and serum creatinine within 3 weeks. At 2- and 8-month follow-ups, serum creatinine had returned to normal (0.75 mg/dL), and proteinuria had resolved. Of note, the patient’s 2 daughters and husband were serologically negative and did not show any symptoms related to a hantavirus infection throughout the initial treatment and follow-up period. We performed those serologic tests 2 weeks after the initial diagnosis of the patient for the 2 daughters and 2 months later for the husband.

Given the unexpected finding of SEOV as causative agent, we initiated an epidemiologic investigation in collaboration with the local public health and veterinary department, which led to the identification of the pet rat breeder. The breeding facility (≈30 m^3^ in size) was located inside a private apartment, and the rats were bred with the intention of being pet animals. During the epidemiologic investigation (and 3 weeks after the patient tested positive for hantavirus IgG), the breeder, her husband, and her daughter also tested positive for hantavirus IgG but could not recall having symptoms suggestive of a hantavirus infection.

**Figure 2:**
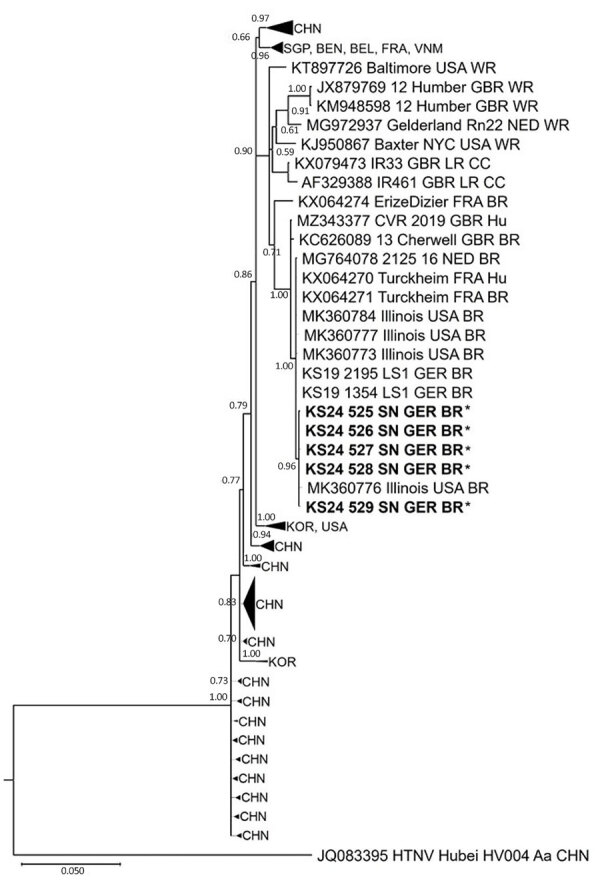
Phylogenetic reconstruction of a partial small segment (731 nt) of Seoul virus isolates (obtained in Saxony state, Germany) in 2024 from rat breeder’s rats implicated in case of severe Seoul virus infection with acute kidney injury, Germany, March 2024. Phylogeny indicates high phylogenetic relatedness among the isolates from the breeder’s rats (bold and asterisks) and other publicly available Seoul virus outbreak strains (*4*,*8*) (Appendix). Scale bar indicates the number of substitutions per site. BEL, Belgium; BEN, Benin; BR, breeder rat; CC, cell culture isolate; CHN, China; FRA, France; GBR, Great Britain; GER, Germany; HU, human; KOR, Korea; NED, the Netherlands; SGP, Singapore; USA, United States; VNM, Vietnam; WR, wild rat.

To assess the source of infection, we also analyzed samples from the index patient’s rats and from the breeding facility. For all 4 of the patient’s rats, we could not determine evidence of SEOV infection by serologic and molecular assays. In contrast, among the 6 pet rats obtained from the breeding facility, all yielded positive results across >1 assays (Appendix Table 2). One rat (source of sample KS24/530) was seropositive but negative by molecular testing, possibly reflecting a low viral load or virus clearance. Another rat (source of sample KS24/528) was SEOV RNA–positive but seronegative, suggesting an acute phase of infection. In general, results were concordant across 4 different assays performed on liver and lung tissues. In addition, we performed a phylogenetic analysis of a 731-nt long region of the viral small segment of the strains obtained from the breeder rats and publicly available SEOV sequences. All strains clustered closely together and shared a relatedness to sequences from other SEOV outbreaks in Germany but also to breeder rats or pet rats from other countries in Europe and the United States (Figure 2). Together with the high sequence similarity of the large segment sequences of 291 nt between the virus detected in the patient and those from the rats of the breeding facility (Appendix Figure 2), those findings strongly suggest the breeding facility as the source of SEOV infection. Given that the pet rats owned by the patient had no evidence of SEOV, the infection probably was acquired through aerosols during a visit to the breeding facility before purchase of the pet rats, followed by a 5-week incubation period until onset of disease symptoms.

## Conclusions

This report underscores several important observations. First, SEOV infection can lead to major clinical disease, including severe acute kidney injury requiring hemodialysis, even in the absence of hemorrhagic fever. Second, the proportion of asymptomatic infection in exposed persons suggests that SEOV infection might be underdiagnosed. Moreover, with the increasing popularity of pet rats, breeding facilities might serve as critical sources for SEOV, warranting enhanced surveillance. Proactive measures, such as routine screening of rat colonies and comprehensive public education, are warranted. 

In Germany, practical implementation of a One Health approach for SEOV must consider the current lack of regulation for private breeders and the effect of animal welfare constraints, which preclude routine invasive testing of asymptomatic animals. Instead, feasible measures should focus on strict hygiene protocols, appropriate ventilation of animal housing, quarantine of newly acquired rats, and thorough documentation of animal origin and health status to facilitate traceability in case of outbreaks (*9*). Given that SEOV is primarily transmitted through inhalation of virus-contaminated aerosols from rodent excreta, risk reduction strategies should include avoiding dry cleaning methods, using damp cleaning techniques, and wearing protective masks during cage maintenance. Immunocompromised persons and other vulnerable groups should be explicitly advised against keeping pet rats. In addition, raising public awareness of zoonotic disease risks through veterinarians, breeders, pet stores, and public health agencies remains essential. Clinicians should routinely inquire about rodent exposures when evaluating patients with unexplained febrile illnesses, particularly when acute kidney injury has occurred (*9*). 

Looking ahead, the development and provision of easy-to-use, noninvasive, and animal welfare–compatible diagnostic tools, such as point-of-care tests for SEOV RNA detection from environmental, oral, or fecal swabs, would offer veterinarians and breeders a practical means of monitoring colonies without causing undue stress to the animals. Such approaches could contribute substantially to early detection and prevention efforts, thereby strengthening One Health strategies in this context.

Appendix Additional information about Seoul virus infection acquired at private pet rat breeding facility, Germany, 2024.
