# Supplementary material for: Seoul Virus Infection Acquired at Private Pet Rat Breeding Facility, Germany, 2024
Source: Emerg Infect Dis. 2025 Oct;31(10):1988–91. doi: 10.3201/eid3110.250362 (PMC12483027; doi:10.3201/eid3110.250362)
Supplement: Appendix — Additional information about Seoul virus infection acquired at private pet rat breeding facility, Germany, 2024. [file 25-0362-Techapp-s1.pdf]

Article DOI: https://doi.org/10.3201/eid3110.250362

EID cannot ensure accessibility for supplementary materials supplied by authors. Readers who have difficulty accessing supplementary content should contact the authors for assistance.

Seoul Virus Infection Acquired at Private Pet Rat Breeding Facility, Germany, 2024

Appendix

Methods

Nucleic Acid Extraction of Rat Specimen

Nucleic acids were isolated from lung and liver tissues of pet rats with QIAzol lysis reagent according to a previously published protocol (*1*).

Hantavirus RT-PCR and Sequence Analysis of Rat Specimen

Conventional Seoul orthohantavirus (SEOV) S and L segment one-step RT-PCR was performed using the SuperScript III RT-PCR Kit (Qiagen) according to protocols described previously (*2*). RT-PCR products of the expected size were sequenced by dideoxy chain termination sequencing with the BigDye Terminator v1.1 Cycle Sequencing Kit (Applied Biosystems, Waltham, MA, USA) according to Sanger. Consensus sequences were created and aligned to determine single nucleotide polymorphisms using BioEdit v7.2.5 (*3*).

In addition, two real-time RT-PCR assays were performed for detection of SEOV RNA. One assay was based on a qRT-PCR using SEOV specific primers (*4*) and the QuantiTect Probe RT-PCR Kit (Qiagen) including an adapted β-actin-specific qRT-PCR as internal PCR control (*5*). In addition, a pan-hantavirus RT-PCR was performed via a SYBR Green-based quantitative real-time RT-PCR assay with primers PanHS8_ Forward CAGGAYATGVGRAAYACVATHATGGC S and PanHS8_Reverse CTCWGGRTCCATRTCATCMCC (*6*) coupled to melting curve analysis using a previously published protocol (*7*).

Phylogenetic Reconstruction of the Partial S-segment (731 nt) and L-segment (291 nt) Sequences

The best-fit substitution model, the generalized time-reversible model (GTR) with gamma distribution and some sites invariable, was determined with JModelTest2 (*8*,*9*), MrBayes v.3.2.7 (*10*) and then used to reconstruct phylogenetic trees with 20 million iterations sampled every 2,000 iterations, and the first 25% discarded as burn-in.

Genbank Accession Numbers

KS24_526_ L_segment_partial PV232940

KS24_528_ L_segment_partial PV232941

KS24_529_ L_segment_partial PV232942

KS24_525_ S_segment_partial PX060514

KS24_526_ S_segment_partial PX060515

KS24_527_ S_segment_partial PX060516

KS24_528_ S_segment_partial PX060517

KS24_529_ S_segment_partial PX060518

SEOV/Leipzig/2024/1 (human) L_segment_partial PV477848

Serologic Investigation of Rat Specimen

Depending on availability, either whole blood samples or chest cavity fluid (CCF: blood diluted in phosphate-buffered saline, PBS) from rats were investigated by an inhouse SEOV-IgG ELISA as described before (*2*) and in parallel by a recomLine HantaPlus IgG assay (Art.-Nr. 7672) from Mikrogen Diagnostics. Blood from SEOV nucleocapsid protein immunized and negative control rats were used as positive and negative controls, respectively.

References

1. Schmidt S, Saxenhofer M, Drewes S, Schlegel M, Wanka KM, Frank R, et al. High genetic structuring of Tula hantavirus. Arch Virol. 2016;161:1135–49. PubMed https://doi.org/10.1007/s00705-016-2762-6

2. Heuser E, Drewes S, Trimpert J, Kunec D, Mehl C, de Cock MP, et al. Pet Rats as the Likely Reservoir for Human Seoul Orthohantavirus Infection. Viruses. 2023;15:467. PubMed https://doi.org/10.3390/v15020467

3. T.A. Hall, BioEdit: a user-friendly biological sequence alignment editor and analysis program for Windows 95/98/NT.

4. Kramski M, Meisel H, Klempa B, Krüger DH, Pauli G, Nitsche A. Detection and typing of human pathogenic hantaviruses by real-time reverse transcription-PCR and pyrosequencing. Clin Chem. 2007;53:1899–905. PubMed https://doi.org/10.1373/clinchem.2007.093245

5. Toussaint JF, Sailleau C, Breard E, Zientara S, De Clercq K. Bluetongue virus detection by two real-time RT-qPCRs targeting two different genomic segments. J Virol Methods. 2007;140:115–23. PubMed https://doi.org/10.1016/j.jviromet.2006.11.007

6. Goodfellow SM, Nofchissey RA, Ye C, Dunnum JL, Cook JA, Bradfute SB. Use of a Novel Detection Tool to Survey Orthohantaviruses in Wild-Caught Rodent Populations. Viruses. 2022;14:682. PubMed https://doi.org/10.3390/v14040682

7. Jonsson CB, Figueiredo LTM, Vapalahti O. A global perspective on hantavirus ecology, epidemiology, and disease. Clin Microbiol Rev. 2010;23:412–41. PubMed https://doi.org/10.1128/CMR.00062-09

8. Guindon S, Gascuel O. A simple, fast, and accurate algorithm to estimate large phylogenies by maximum likelihood. Syst Biol. 2003;52:696–704. PubMed https://doi.org/10.1080/10635150390235520

9. Darriba D, Taboada GL, Doallo R, Posada D. jModelTest 2: more models, new heuristics and parallel computing. Nat Methods. 2012;9:772. PubMed https://doi.org/10.1038/nmeth.2109

10. Ronquist F, Teslenko M, van der Mark P, Ayres DL, Darling A, Höhna S, et al. MrBayes 3.2: efficient Bayesian phylogenetic inference and model choice across a large model space. Syst Biol. 2012;61:539–42. PubMed https://doi.org/10.1093/sysbio/sys029

11. Hofmann J, Ulrich RG, Mehl C, Drewes S, Esser J, Loyen M, et al. Hantavirus Disease Cluster Caused by Seoul Virus, Germany. Emerg Infect Dis. 2024;30:133–5. PubMed https://doi.org/10.3201/eid3001.230855

12. Kramski M, Meisel H, Klempa B, Krüger DH, Pauli G, Nitsche A. Detection and typing of human pathogenic hantaviruses by real-time reverse transcription-PCR and pyrosequencing. Clin Chem. 2007;53:1899–905. PubMed https://doi.org/10.1373/clinchem.2007.093245

13. Goodfellow SM, Nofchissey RA, Ye C, Dunnum JL, Cook JA, Bradfute SB. Use of a novel detection tool to survey orthohantaviruses in wild-caught rodent populations. Viruses. 2022;14:682. PubMed https://doi.org/10.3390/v14040682

14. Haake AFH, Eisenberg T, Heuser E, Stollberg KC, Richter MH, Dreesman J, et al. Cuddle with care! A current overview of zoonotic pathogens transmitted by pet rats. Berl Munch Tierarztl Wochenschr. 2024;137:1–17. https://doi.org/10.2376/1439-0299-2024-7

Appendix Table 1. Antibody reactivity of patient blood samples for IgG and IgM (recomLine HantaPlus)*

Days after Admission	Antibody class	Puumala virus	Sin Nombre virus	Hantaan virus	Dobrava virus	Seoul virus	SFFV
1	IgG	–	–	**1.6**	0.6	–	–
	IgM	**3.3**	0.6	**11.2**	**11.5**	**11.3**	0.5
19	IgG	–	–	**4.1**	**1.9**	0.8	–
	IgM	**1.1**	0.4	**7.4**	**8.2**	**7.8**	–
54	IgG	0.6	–	**6.2**	**3.6**	**3.2**	–
	IgM	–	0.4	**1.6**	**1.7**	**1.7**	–

*IgG and IgM Assays were performed with *recom*Line HantaPlus lineblots on an automated Car*l* platform (all Mikrogen Diagnostics, Neuried, Germany) according to the manufacturer's instructions. The band intensity for the different hantavirus antigens and the Sandfly fever virus (SFFV) was assessed using the the *recom*Scan software with an intensity of 1,0 being equivalent to the cutoff band.

Appendix Table 2. Summary of results from serologic and molecular assays of rat samples from patient and breeding facility

Sample ID	Sex	Weight, g	IgG ELISA	IgG RecomLine blot	Result serology	L RT-PCR	S RT-PCR (*12*)	Sequence	SEOV qRT-PCR (*12*)	Pan-hantavirus qRT-PCR (*13*)	Result molecular biology
KS24/489*	F	237.9	Neg	Neg	Neg	Neg	Neg	NA	Neg	Neg	Neg
KS24/490*	F	232.1	Neg	Neg	Neg	Neg	Neg	NA	Neg	Neg	Neg
KS24/491*	F	217.0	Neg	Neg	Neg	Neg	Neg	NA	Neg	Neg	Neg
KS24/492*	F	255.6	Neg	Neg	Neg	Neg	Neg	NA	Neg	Neg	Neg
KS24/525#	F	223.1	Pos	Pos	Pos	Neg	Pos	S: 732 nt	Neg	Pos	Pos
KS24/526#	F	332.5	Pos	Weakly pos	Weakly pos	Pos	Pos	S: 732 nt, L: 412 nt	Pos	Pos	Pos
KS24/527#	F	239.5	Pos	Pos	Pos	Pos	Weakly pos	S: 732 nt, L: 341 nt	Neg	Pos	Pos
KS24/528#	M	492.0	Neg	Neg	Neg	Pos	Pos	S: 732 nt, L: 412 nt	Pos	Pos	Pos
KS24/529#	M	412.5	Pos	Pos	Pos	Pos	Pos	S: 732 nt; L: 412 nt	Pos	Pos	Pos
KS24/530#	M	164.3	Pos	Pos	Pos	Neg	Neg	NA	Neg	Neg	Neg

Samples KS24/489–492 (*, chest cavity fluid, lung and liver tissue) were obtained from the patient's rats, while samples KS23/525–530 (#, chest cavity fluid and lung tissue) originated from the breeding facility (all Species Rattus norvegicus). The assays include ELISA and recomLine (Mikrogen Diagnostics) for the detection of hantavirus IgG antibodies performed with chest cavity fluid. Conventional RT-PCR of tissue samples targets the viral large (L) and small (S) segment (*14*). Additional molecular detection was performed using qRT-PCR and a modified pan-orthohantavirus qRT-PCR (*12,13*) (for details see Appendix). Abbreviation: ELISA, enzyme-linked immunosorbent assay; L, large; neg, negative; n.a., not applicable; nt, nucleotides; PCR, polymerase chain reaction; pos, positive; Rattus n., Rattus norvegicus; RT-PCR, reverse transcription PCR; qRT-PCR, quantitative RT-PCR; S, small; SEOV, Seoul virus; F, female; M, male

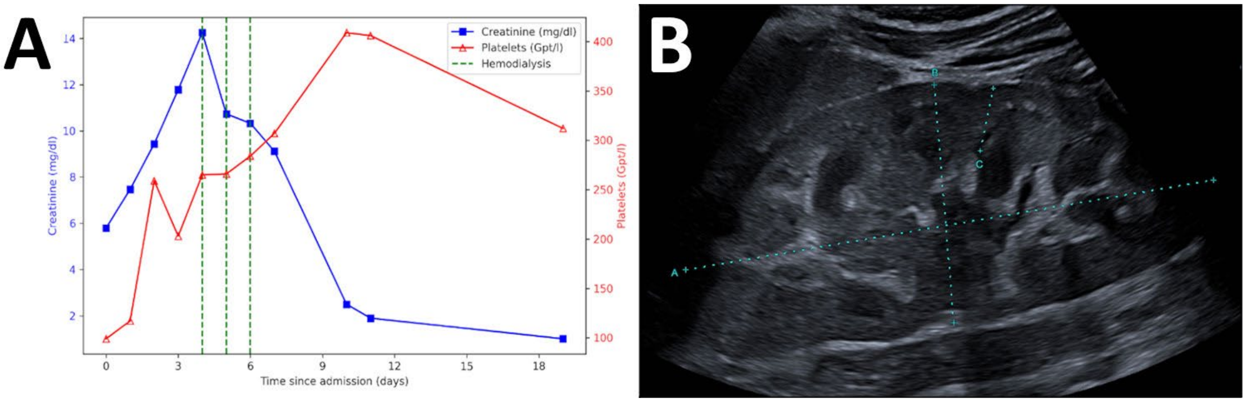

Appendix Figure 1. A) The index patient's laboratory work-up shows progressive acute kidney injury with rising serum creatinine (blue) and concomitant thrombopenia (red). On day 4 after admission the patient required hemodialysis (green). Kidney function started to regenerate on day 7 after admission, obviating further dialysis. B) Sonography of the left kidney reveals a swollen organ, consistent with acute kidney injury (A length: 14.3 cm, B width: 6.3 cm, C parenchymal thickness: 1.7 cm).

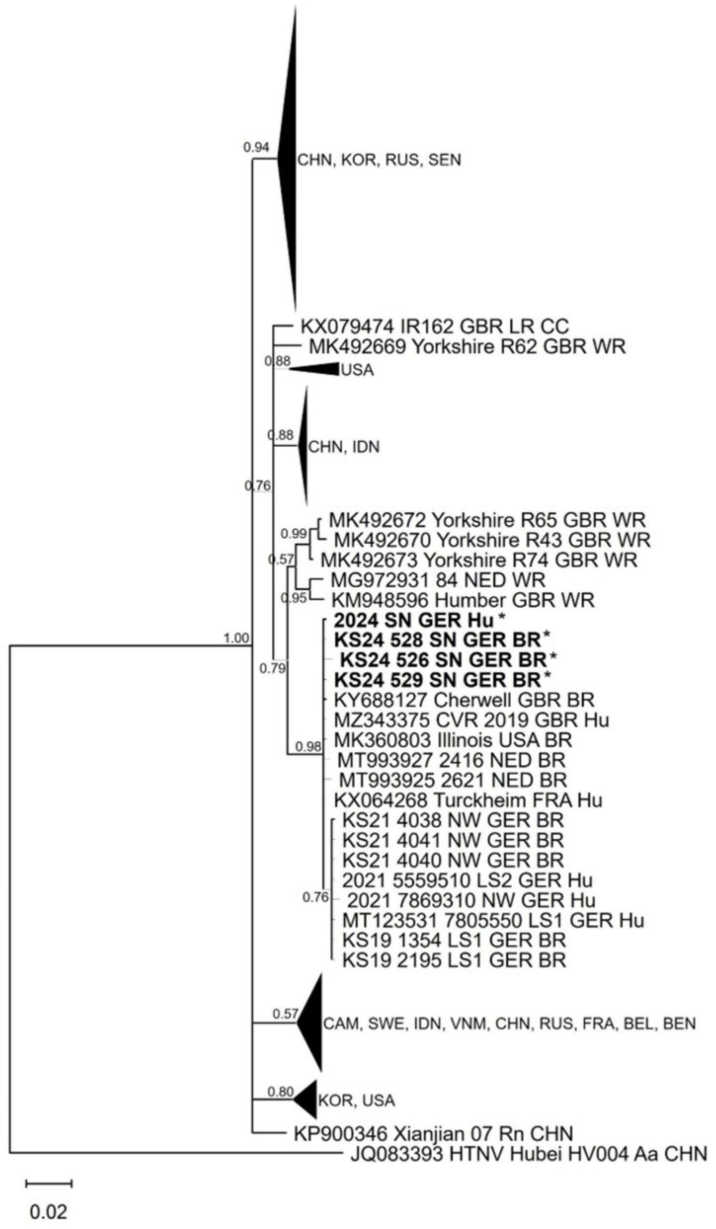

Appendix Figure 2. Phylogenetic reconstruction of a partial L-segment (291 nt) of the rat breeder's rats and the patient show a high phylogenetic relatedness toward each other, and to other publicly available SEOV outbreak strains. Sequences derived from the described breeder rats and the patient are marked by an asterisk and the corresponding federal state (Saxony, SN). The other breeder rat- and patient derived sequences are also labeled with the federal state abbreviations (LS, Lower Saxony; NW, North Rhine-Westphalia; SN), following the description by Heuser et al and Hofmann et al (*2*,*11*). Country abbreviations: BEL, Belgium; BEN, Benin; CHN, People's Republic of China; FRA, France; GBR, Great Britain; GER, Germany; KOR, Korea; NED, the Netherlands; SGP, Singapore; USA, United States of America; VNM, Vietnam. Other abbreviations: HU, human; BR, breeder rat; CC, cell culture isolate; WR, wild rat.
